# Editorial Comment to “Daytime Bladder Control Status in Toddlerhood Is Associated With Subsequent Bedwetting in Preschool Years: A Nationwide Cohort Study of Over 30 000 Japanese Children”

**DOI:** 10.1111/iju.70333

**Published:** 2025-12-29

**Authors:** Kenichi Kobayashi

**Affiliations:** ^1^ The Department of Urology at Shiga University of Medical Science Otsu Shiga Japan

The possibility that early‐life urinary habits and developmental patterns may influence later urinary function, including the persistence of bedwetting and voiding dysfunction, has been discussed for many years [1, 2]. However, according to the International Children's Continence Society (ICCS), nocturnal enuresis (NE) is defined as urinary incontinence during sleep in children aged 5 years or older [3]. Therefore, there has been little solid data or evidence regarding urinary symptoms or nighttime wetting before this age.

In this context, the present study provides valuable and timely insights using a large‐scale national cohort that offers robust and reliable data [4].

The authors examined daytime bladder control at 2.5 years of age, bedwetting at 4.5 years, and the relationship between these two factors. They found that 82.8% of children were still using diapers at 2.5 years, and that the prevalence of bedwetting at 4.5 years was 42.2%. These findings, based on high‐quality longitudinal data, shed light on an important but underexplored developmental stage that precedes the conventional definition of enuresis. Moreover, the authors demonstrated that incomplete daytime bladder control at 2.5 years was associated with an increased risk of the persistence of bedwetting.

The data were drawn from the “Longitudinal Survey of Newborns in the 21st Century,” a nationwide birth cohort conducted by the Ministry of Health, Labour and Welfare in Japan, including more than 30 000 children. The use of such a large, well‐constructed dataset is one of the major strengths of this study and strongly supports the reliability of its findings. As mentioned earlier, studies focusing on urinary control at this early age are extremely limited. Therefore, this study makes a highly valuable contribution simply by providing reliable data in this field.

Readers should remember that this is an observational study. The assessment of bladder control was based on diaper use, which reflects the actual use of diapers rather than a direct measurement of bladder function. The study does not address how diaper use itself might influence urinary control development. This is an important point to keep in mind when interpreting the findings.

Overall, this study provides a unique and informative perspective on early childhood urinary development. It highlights the importance of understanding how early‐life bladder control may relate to later urinary symptoms, and it offers a strong foundation for future longitudinal or interventional research in this area.

## Author Contributions

The author takes full responsibility for this article.

## Conflicts of Interest

The author declares no conflicts of interest.

## Linked Articles

This article is linked to Moriwake et al. papers. To view this article, visit https://doi.org/10.1111/iju.70288.
